# Association between tranexamic acid and postoperative delirium in elderly patients: a mediation analysis of inflammatory markers following hip and knee arthroplasty

**DOI:** 10.3389/fmed.2026.1865593

**Published:** 2026-07-31

**Authors:** Junyang Chu, Lin Gao, Lei Xie, Yushun Liu, Yixuan Zhu, Kang Deng

**Affiliations:** 1 Zhejiang Chinese Medical University, Hangzhou, China; 2 Department of Anesthesiology and Pain Medicine, Affiliated Hospital of Jiaxing University, Jiaxing, China; 3 Jiaxing University, Jiaxing, China

**Keywords:** arthroplasty, elderly, inflammation, postoperative delirium, tranexamic acid

## Abstract

**Background:**

Postoperative delirium (POD) is a common complication in elderly patients undergoing hip or knee arthroplasty, and neuroinflammation plays a critical role in its pathogenesis. In addition to its antifibrinolytic properties, tranexamic acid (TXA) also exhibits anti-inflammatory effects. However, whether there is an association among TXA, inflammation, and POD remains poorly investigated. Therefore, this study aimed to investigate the effect of intravenous TXA on the incidence of POD in elderly patients undergoing hip or knee arthroplasty and to analyze the potential mediating role of inflammatory markers.

**Methods:**

This single-center, retrospective cohort study included 1,234 elderly patients (aged ≥65 years) undergoing hip or knee arthroplasty at our institution between January 2017 and January 2026. Patients were divided into the TXA group (*n* = 651) and the control group (n = 583) based on the intraoperative administration of intravenous TXA. Outcomes included POD incidence within 7 days and 48-h postoperative markers: neutrophil-to-lymphocyte ratio (NLR), systemic inflammation response index (SIRI), monocyte-to-lymphocyte ratio (MLR), systemic immune-inflammation index (SII), platelet-to-lymphocyte ratio (PLR), high-sensitivity C-reactive protein (hs-CRP), and interleukin-6 (IL-6). Multivariable logistic regression, linear regression, and subgroup analyses were performed to evaluate the associations, while mediation analysis was conducted to assess the potential role of inflammatory markers.

**Results:**

The incidence of POD was significantly lower in the TXA group than in the control group (26.0% vs. 41.9%, *p* < 0.001). Additionally, postoperative levels of all inflammatory markers (NLR, SIRI, MLR, SII, PLR, hs-CRP, and IL-6) were significantly reduced in the TXA group (*p* < 0.001). Adjusting for confounders, TXA remained an independent protective factor against POD (OR = 0.415, 95% CI: 0.305, 0.563, *p* < 0.001). Exploratory mediation analysis showed that NLR, SIRI, SII, hs-CRP, and IL-6 may partially mediate the association between TXA and a reduced risk of POD, with mediation proportions of 18.17, 9.88, 14.75, 14.23, and 5.56%, respectively. Subgroup analyses confirmed that intravenous TXA remained a protective factor against POD across various patient strata.

**Conclusion:**

Intravenous TXA administration is significantly associated with a reduced incidence of POD in elderly patients undergoing hip or knee arthroplasty. This association may be partially mediated by inflammatory markers, including NLR, SIRI, SII, hs-CRP, and IL-6. These findings contribute to the development of more effective strategies to improve the quality of postoperative recovery in elderly patients.

## 1 Introduction

With the global rise in the aging population and obesity rates, the incidence of hip and knee osteoarthritis (OA) has been steadily increasing (1). This condition, largely driven by increased mechanical loading on joints, primarily manifests as joint pain and functional impairment. Hip arthroplasty and knee arthroplasty are widely recognized as effective interventions for end-stage hip and knee osteoarthritis. Statistics indicate that approximately 512,000 THA and 700,000 TKA procedures are performed annually in the United States. In China, the total volume of these surgeries reached 1 million in 2021 and is projected to continue rising (2–4). Postoperative delirium (POD) is a common complication following hip and knee arthroplasty in elderly patients, typically characterized by cognitive impairment, behavioral abnormalities, emotional fluctuations, and disturbed sleep–wake cycles. Previous studies have reported POD incidences as high as 61.0 and 19.1% in elderly patients undergoing hip and knee arthroplasty, respectively (5, 6). POD is associated with diminished quality of life, increased mortality, prolonged hospital stays, and elevated healthcare costs (7).

Tranexamic acid (TXA) is a synthetic analog of the amino acid lysine. It exerts its antifibrinolytic effect by reversibly blocking the lysine-binding sites on plasminogen molecules, thereby inhibiting fibrin degradation and reducing blood loss. Due to its potent hemostatic properties, TXA is widely utilized in surgical procedures. Previous studies have confirmed that both intravenous and oral administration effectively reduce intraoperative blood loss and transfusion requirements (8). In recent years, the anti-inflammatory effects of TXA in clinical practice have gained increasing recognition. Evidence suggests that TXA administration effectively reduces the levels of inflammatory cytokines following total hip arthroplasty, total knee arthroplasty, and cardiac surgery (9–11). While the precise anti-inflammatory mechanisms of TXA remain to be fully elucidated, existing studies suggest that its effects may be related to the inhibition of plasmin and complement activation, as well as the suppression of pro-inflammatory cytokine expression (12, 13).

The pathogenesis of POD remains poorly understood; however, advances in research into its pathophysiological mechanisms suggest a multifactorial etiology. Studies have demonstrated that the systemic inflammatory response and oxidative stress injury induced by surgery are closely associated with the occurrence of POD (14, 15). In recent years, hemogram-based inflammatory parameters including NLR, systemic inflammation response index (SIRI), MLR, systemic immune-inflammation index (SII), and PLR have gained increasing attention as effective predictors of POD (16, 17). Furthermore, evidence indicates that suppressing inflammatory responses can effectively reduce the incidence of POD (18, 19). These findings suggest that both POD and TXA are closely linked to inflammatory responses. Therefore, we hypothesize that TXA may influence the occurrence of POD in elderly patients by modulating systemic inflammation levels; however, research in this area remains insufficient. We conducted a retrospective study to investigate the impact of intravenous TXA on the incidence of POD in elderly patients undergoing hip or knee arthroplasty and to evaluate whether inflammatory markers mediate the association between TXA and POD.

## 2 Materials and methods

### 2.1 Study design and participants

**Figure 1:**
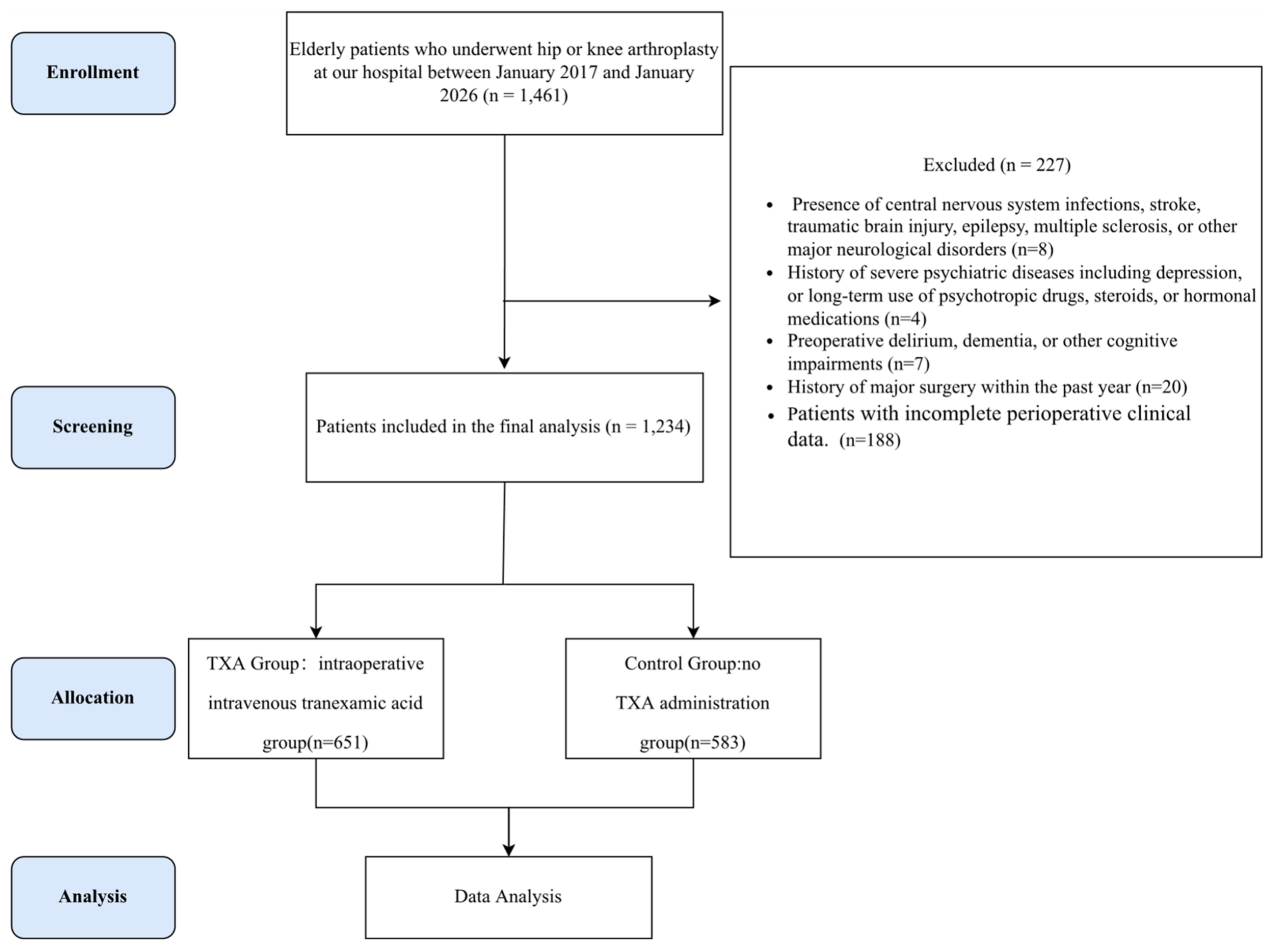
Flow diagram of participants included in the study.

This single-center, retrospective cohort study enrolled 1,461 patients who underwent hip or knee arthroplasty at the Department of Orthopedics, Affiliated Hospital of Jiaxing University, between January 2017 and January 2026. After applying inclusion and exclusion criteria, 1,234 patients were ultimately included in the analysis. Patients were categorized into two groups based on whether intravenous TXA was administered intraoperatively: the TXA group (received TXA) and the control group (did not receive TXA). The inclusion criteria were as follows: (1) age ≥ 65 years, (2) undergoing hip or knee arthroplasty, (3) American Society of Anesthesiologists (ASA) physical status I–III, and (4) receiving general anesthesia. The exclusion criteria were as follows: (1) presence of central nervous system infections, stroke, traumatic brain injury, epilepsy, multiple sclerosis, or other major neurological disorders; (2) history of severe psychiatric diseases including depression, or long-term use of psychotropic drugs, steroids, or hormonal medications; (3) preoperative delirium, dementia, or other cognitive impairments; (4) history of major surgery within the past year; and (5) patients with incomplete perioperative clinical data. The participant selection process is illustrated in Figure 1.

This retrospective study was approved by the Ethics Committee of the Affiliated Hospital of Jiaxing University (Approval No. 2026-LP-044) and registered with the Chinese Clinical Trial Registry (ChiCTR2600118722). All procedures were conducted in accordance with the principles of the Declaration of Helsinki. The requirement for informed consent was waived by the Ethics Committee. All data were retrieved from a de-identified electronic medical record system to ensure patient privacy and confidentiality.

### 2.2 Data collection

Study data were extracted from electronic medical record and postoperative follow-up systems. We recorded comprehensive perioperative variables to be used as potential covariates, including age, gender, BMI, ASA grade, smoking history, alcohol consumption history, preoperative comorbidities (hypertension, diabetes, CHD, COPD, and pneumonia), educational level, and MoCA score. Additionally, the type of surgery and operative time were documented. Perioperative hemoglobin (Hb) levels, preoperative renal function, intraoperative blood loss, intraoperative blood transfusion volume, occurrence of intraoperative hypotension (defined as systolic blood pressure <90 mmHg or mean arterial pressure <65 mmHg lasting for more than 5 min), 24-h postoperative Numerical Rating Scale (NRS) pain score, intraoperative and postoperative opioid consumption, and the occurrence of postoperative adverse events, including lower-extremity deep venous thrombosis (LEDVT), pulmonary embolism (PE), cerebral infarction, myocardial infarction, and ICU admission.

Preoperative cognitive status was assessed by doctors 1 day prior to surgery using the Montreal Cognitive Assessment (MoCA). The MoCA scale provides a comprehensive evaluation of multiple cognitive domains, including executive function, memory, language, visuospatial skills, attention, and working memory. The total score is 30 points, with higher scores indicating better cognitive function. A score of 26 or higher is defined as normal cognitive function (20).

### 2.3 Anesthesia management

All patients were fasted as required. Upon arrival in the operating room, BP, ECG, HR, SpO_2_, RR, and BIS were monitored. General anesthesia was induced with etomidate (0.25 mg/kg), sufentanil (0.3 μg/kg), and cisatracurium (0.15 mg/kg). Endotracheal intubation was performed following the loss of eyelash reflex and verbal response with a BIS < 60. Mechanical ventilation was used in PCV-VG mode: tidal volume 6–8 mL/kg, RR 12–20 breaths/min, and ETCO_2_ 30–40 mmHg. Anesthesia maintenance involved continuous infusion of propofol (2–4 mg·kg^−1^·h^−1^) and remifentanil (0.02–0.20 μg·kg^−1^·min^−1^), adjusted to maintain a BIS of 40–60. Patients were transferred to the PACU postoperatively. The endotracheal tube was removed once spontaneous respiration was restored, and the patients returned to the ward upon regaining full consciousness. Postoperative analgesia was provided using patient-controlled intravenous analgesia (PCIA) for all patients, with sufentanil as the analgesic agent.

TXA Administration Protocol: A measure of 1.5 g of tranexamic acid was dissolved in 100 mL of 0.9% sodium chloride solution and administered via intravenous infusion at the initiation of surgery, which was completed within 20 min.

### 2.4 Postoperative delirium assessment

Data regarding psychiatric status were retrieved from the patients’ electronic medical records. POD was diagnosed using the Confusion Assessment Method (CAM) based on the following clinical criteria: (1) acute onset and fluctuating course: sudden onset with significant fluctuations; (2) inattention: difficulty maintaining persistent attention or reduced responsiveness to external stimuli; (3) disorganized thinking: fragmented or illogical thought processes and speech; and (4) altered level of consciousness: fluctuations in alertness, ranging from lethargy to hyperalertness. A diagnosis of POD requires the presence of both features (1) and (2), plus either feature (3) or (4).

POD was assessed by clinicians trained in neuropsychology using the Confusion Assessment Method. Assessments were routinely conducted two times daily, scheduled at 08:00–10:00 and 16:00–18:00. Additional assessments were performed immediately if patients exhibited altered consciousness, disorganized thinking, or other abnormal behaviors.

### 2.5 Measurement of blood inflammatory markers and their ratios

Fasting peripheral venous blood samples (5 mL) were collected from all participants 48 h after surgery. Serum was separated using non-anticoagulant vacuum tubes and centrifuged at 1500 × g for 5 min. High-sensitivity C-reactive protein (hs-CRP) levels were measured via latex-enhanced immunoturbidimetry using an automatic biochemical analyzer (C16000; Abbott, Abbott Park, IL, USA), with reagents provided by Zhejiang Quark Biotechnology Co., Ltd. (Zhejiang, China). Interleukin-6 (IL-6) levels were determined using a double-antibody sandwich acridinium ester direct chemiluminescence immunoassay on an automatic chemiluminescence immunoassay analyzer (Caris200; Xiamen UMIC Medical Co., Ltd., Xiamen, China), with reagents purchased from Xiamen Wantai Canghai Biotechnology Co., Ltd. (Xiamen, China). Additionally, 2 mL of peripheral blood was collected using ethylenediaminetetraacetic acid dipotassium (EDTA-K_2_) anticoagulant tubes. Neutrophil, platelet, monocyte, and lymphocyte counts were determined using a Sysmex-XE2100 automatic hematology analyzer (Sysmex Corp., Kobe, Japan). To ensure the authenticity and reliability of the findings, all data were independently cross-checked and verified by two experienced clinicians before being entered for analysis. In light of evidence from previous studies establishing their association with POD, we selected several blood inflammatory markers for this study (16, 17, 21, 22). These markers included NLR, SIRI, MLR, SII, PLR, hs-CRP, and IL-6. The markers were calculated as follows: NLR (neutrophil/lymphocyte), SIRI (neutrophil × monocyte/lymphocyte), MLR (monocyte/lymphocyte), SII (platelet × neutrophil/lymphocyte), and PLR (platelet/lymphocyte). hs-CRP and IL-6 were measured directly.

### 2.6 Covariates

Based on literature and clinical relevance, several covariates were included: age, gender, BMI, ASA grade, smoking history, alcohol consumption history, preoperative comorbidities (hypertension, diabetes mellitus, CHD, COPD, and pneumonia), educational level (junior high school or below and senior high school or above), MoCA score, type of surgery (hip arthroplasty and knee arthroplasty), operative time, and preoperative inflammatory markers (NLR, SIRI, MLR, SII, PLR, hs-CRP, and IL-6). BMI was calculated as weight (kg) divided by the square of height (m^2^).

### 2.7 Sample size calculation

The sample size was calculated based on the primary endpoint, postoperative delirium. In a preliminary retrospective analysis, we included 150 patients in the TXA group (receiving intraoperative TXA) and 150 patients in the control group (not receiving TXA). Based on a preliminary analysis, POD incidence was 26.5% in the TXA group and 35.2% in the control group. The sample size was estimated using PASS 15.0. With *α* = 0.05 (two-sided), 80% power (*β* = 0.20), and a 1:1 ratio, 439 patients per group (878 total) were required. After adjusting for a 20% exclusion rate, the minimum requirement was 549 per group (1,098 total). Our final inclusion of 1,234 participants satisfied the required sample size for statistical power.

### 2.8 Statistical analysis

Normally distributed continuous variables are expressed as mean ± SD, while non-normally distributed variables are presented as median (IQR: Q1, Q3). Categorical variables are reported as frequencies (percentages). Differences between the TXA and control groups were compared using independent-samples *t*-tests, chi-squared tests, and Mann–Whitney U-tests, as appropriate. Binary logistic regression was used to evaluate the association between intravenous TXA and POD incidence through three models: Model 1 was unadjusted; Model 2 was adjusted for gender, age, BMI, ASA grade, smoking history, alcohol consumption history, educational level, MoCA score, and preoperative comorbidities; Model 3 was adjusted for age, sex, BMI, ASA classification, smoking history, alcohol consumption history, education level, MoCA score, preoperative comorbidities, type of surgery, operative duration, preoperative inflammatory marker levels, preoperative and postoperative Hb levels, preoperative renal function, intraoperative blood loss, intraoperative blood transfusion volume, occurrence of intraoperative hypotension, 24-h postoperative NRS pain score, and intraoperative and postoperative opioid consumption. The results are presented as OR and 95% CI. Furthermore, regression analysis was used to evaluate the associations between the seven inflammatory markers (NLR, SIRI, MLR, SII, PLR, hs-CRP, and IL-6) and both TXA administration and POD.

Subgroup analyses were further conducted to assess the consistency of the association between TXA administration and POD incidence across different populations. These subgroups were defined by gender, age, BMI, educational level, MoCA score, ASA grade, type of surgery, smoking history, alcohol consumption history, preoperative complications, and operative time.

Given the observational design of this study, the mediation analysis was considered exploratory and was intended to generate research hypotheses. Exploratory mediation analysis based on the bootstrap method was performed to examine the potential mediating roles of NLR, SIRI, SII, hs-CRP, and IL-6 in the association between TXA use and the occurrence of POD. To enhance the accuracy of the results, potential confounding variables were adjusted according to Model 3. All mediation and subgroup analyses were performed using the fully adjusted Model 3. Mediation analysis was performed using the SPSS PROCESS macro (version 4.2). Indirect effects were estimated via bootstrapping with 5,000 resamples to construct 95% CI.

Statistical significance was set at a *p*-value of < 0.05. Statistical analyses were performed using IBM SPSS (version 29.0).

## 3 Result

### 3.1 Study population and baseline characteristics

A total of 1,234 patients (804 females and 430 males) aged over 65 years were included according to the inclusion and exclusion criteria. The mean age of the cohort was 74.84 ± 7.14 years. Patients were divided into the TXA group (n = 651) and the control group (n = 583) based on the intraoperative intravenous administration of TXA. Baseline characteristics are summarized in Table 1. Intraoperative blood loss was lower in the TXA group than in the control group. The 24-h postoperative NRS pain score and intraoperative remifentanil consumption also differed between the two groups (all *p* < 0.05). No significant differences were observed between the two groups in age, sex, BMI, ASA classification, smoking history, alcohol consumption history, education level, MoCA score, preoperative comorbidities, preoperative inflammatory marker levels, preoperative and postoperative Hb levels, preoperative renal function, intraoperative blood transfusion volume, incidence of intraoperative hypotension, intraoperative and postoperative sufentanil consumption, occurrence of postoperative adverse events, or ICU admission rate (*p* > 0.05).

**Table 1:** Baseline characteristics of the study population.

Items	TXA group (*n* = 651)	Control group (*n* = 583)	*P*
Age (years), mean ± SD	74.59 ± 6.95	75.11 ± 7.34	0.210
Gender, *n* (%)			0.462
Male	233(35.8%)	197(33.8%)	
Female	418 (64.2%)	386 (66.2%)	
BMI (kg/m2), IQR	24.01 (21.31, 26.04)	23.81 (20.96, 25.63)	0.183
ASA grade, *n* (%)			0.321
II	340 (52.2%)	288 (49.4%)	
III	311 (47.8%)	295 (50.6%)	
Smoking history, *n* (%)	43 (6.6%)	34 (5.8%)	0.575
Alcohol consumption history, *n* (%)	72 (11.1%)	53 (9.1%)	0.252
Preoperative comorbidity, *n* (%)			
Hypertension	354 (54.4%)	339 (58.1%)	0.183
Diabetes	119 (18.3%)	120 (20.6%)	0.307
CHD	86 (13.2%)	82 (14.1%)	0.662
COPD	86 (13.2%)	86 (14.8%)	0.435
Pneumonia	71 (10.9%)	63 (10.8%)	0.955
Educational level, *n* (%)			0.953
Junior high school or below	364 (55.9%)	325 (55.7%)	
Senior high school or above	287 (44.1%)	258 (44.3%)	
MoCA, IQR	28.00 (27.00,29.00)	28.00 (27.00,29.00)	0.834
Type of surgery, *n* (%)			0.975
Hip arthroplasty	445 (68.4%)	399 (68.4%)	
Knee arthroplasty	206 (31.6%)	184 (31.6%)	
Operative time (min), IQR	120.00 (95.00–140.00)	115.00 (90.00–140.00)	0.159
Preoperative laboratory indicators			
NLR, IQR	3.70 (2.48, 5.95)	3.69 (3.23, 6.29)	0.448
SIRI, IQR	1.35 (0.84, 2.67)	1.49 (0.84, 2.91)	0.373
MLR, IQR	0.31 (0.21, 0.49)	0.30 (0.20, 0.49)	0.387
SII, IQR	684.50 (425.50, 1057.00)	727.50 (426.00, 1190.40)	0.206
PLR, IQR	155.45 (115.38, 206.36)	158.13 (115.00, 212.50)	0.416
hs-CRP (mg/L), IQR	2.80 (0.90, 8.20)	3.20 (0.30, 28.10)	0.448
IL-6 (pg/mL), IQR	3.29 (1.50, 9.26)	3.63 (2.13, 6.41)	0.179
Hemoglobin (g/L), IQR	128 (117, 138)	128 (118, 137)	0.957
Cr (μmol/L), IQR	68.3 (58.0, 81.5)	67.0 (58.3, 78.3)	0.183
BUN(mmol/L), IQR	5.79 (4.71, 6.98)	5.68 (4.63, 6.86)	0.394
Intraoperative variables			
Intraoperative blood loss (mL), IQR	100.00 (50.00, 150.00)	100.00 (50.00, 200.00)	0.016
Intraoperative transfusion (mL), IQR	0.00 (0.00, 0.00)	0.00 (0.00, 0.00)	0.128
Intraoperative sufentanil consumption (μg), IQR	20 (19, 24)	20 (19, 24)	0.499
Intraoperative remifentanil consumption (μg), IQR	1.0 (0.8, 1.1)	1.0 (0.8, 1.1)	0.018
Intraoperative hypotension, n (%)	194 (29.8%)	176 (30.2%)	0.882
Postoperative variables			
Hemoglobin (g/L), IQR	105 (92, 115)	104 (93, 115)	0.960
NRS, IQR	2 (2, 2)	2 (2, 3)	<0.001
Postoperative sufentanil consumption (μg), IQR	92 (72, 100)	92 (64, 100)	0.963
ICU admission, *n* (%)	78 (12.0%)	79 (13.6%)	0.409
Adverse event			
LEDVT, *n* (%)	162 (24.9%)	140 (24.0%)	0.722
PE, *n* (%)	3 (0.5%)	5 (0.9%)	0.487
CI, *n* (%)	4 (0.6%)	2 (0.3%)	0.690
MI, *n* (%)	2 (0.3%)	3 (0.5%)	0.672

BMI, body mass index; ASA, American Society of Anesthesiologists; CHD, coronary heart disease; COPD, chronic obstructive pulmonary disease; MoCA, Montreal Cognitive Assessment; NLR, neutrophil-to-lymphocyte ratio; SIRI, systemic inflammation response index; MLR, monocyte-to-lymphocyte ratio; SII, systemic immune-inflammation index; PLR, platelet-to-lymphocyte ratio; hs-CRP, high-sensitivity C-reactive protein; IL-6, interleukin-6; Cr, creatinine; BUN, blood urea nitrogen; NRS, numeric rating scale; LEDVT, lower extremity deep vein thrombosis; PE, pulmonary embolism; CI, cerebral infarction; MI, myocardial infarction; TXA group, intravenous tranexamic acid group; control group, no intravenous tranexamic acid group.

### 3.2 Incidence of POD and postoperative inflammatory marker levels

The incidence of POD was significantly lower in the TXA group than in the control group [169/651 (26.00%) vs. 244/583 (41.85%), *p* < 0.001]. Regarding postoperative inflammatory markers, the TXA group exhibited significantly lower levels than the control group across all indices: NLR [7.50 (5.40, 10.26) vs. 10.25 (6.38, 13.50), *p* < 0.001], SIRI [3.65 (2.33, 5.74) vs. 4.93 (2.70, 7.85), *p* < 0.001], MLR [0.51 (0.38, 0.69) vs. 0.60 (0.41, 0.86), *p* < 0.001], SII [1237.50 (793.80, 1813.78) vs. 1676.89 (1011.43, 2525.89), *p* < 0.001], PLR [184.29 (137.33, 237.50) vs. 207.86 (154.29, 280.00), p < 0.001], hs-CRP [29.80 (17.20, 47.00) vs. 41.29 (22.60, 60.40), p < 0.001], and IL-6 [63.22 (36.50, 108.39) vs. 82.52 (58.82, 132.60), p < 0.001] (Table 2).

**Table 2:** Comparison of postoperative delirium incidence and postoperative inflammatory markers between the groups.

Items	TXA group (*n* = 651)	Control group (*n* = 583)	*P*
POD, *n* (%)	169 (26.0%)	244 (41.9%)	<0.001
Postoperative inflammatory markers			
NLR, IQR	7.50 (5.40, 10.26)	10.25 (6.38, 13.50)	<0.001
SIRI, IQR	3.65 (2.33, 5.74)	4.93 (2.70, 7.85)	<0.001
MLR, IQR	0.51 (0.38, 0.69)	0.60 (0.41, 0.86)	<0.001
SII, IQR	1237.50 (793.80, 1813.78)	1676.89 (1011.43, 2525.89)	<0.001
PLR, IQR	184.29 (137.33, 237.50)	207.86 (154.29, 280.00)	<0.001
hs-CRP (mg/L), IQR	29.80 (17.20, 47.00)	41.29 (22.60, 60.40)	<0.001
IL-6 (pg/mL), IQR	63.22 (36.50, 108.39)	82.52 (58.82, 132.60)	<0.001

POD, postoperative delirium; NLR, neutrophil-to-lymphocyte ratio; SIRI, systemic inflammation response index; MLR, monocyte-to-lymphocyte ratio; SII, systemic immune-inflammation index; PLR, platelet-to-lymphocyte ratio; hs-CRP, high-sensitivity C-reactive protein; IL-6, interleukin-6; TXA group, intravenous tranexamic acid group; Control group, no intravenous tranexamic acid group.

### 3.3 Association between TXA and POD

Binary logistic regression analysis was used to verify the association between intravenous TXA administration and the occurrence of POD in elderly patients undergoing hip and knee arthroplasty. The results indicated that TXA administration was a protective factor against POD (*p* < 0.05). The OR (95% CI) for TXA administration across Model 1, Model 2, and Model 3 was 0.487 (0.383, 0.619), 0.484 (0.379, 0.618), and 0.415(0.305, 0.563), respectively (Table 3).

**Table 3:** Association between tranexamic acid and postoperative delirium.

Variables	OR	95% CI	*P*
Model 1	0.487	(0.383, 0.619)	<0.001
Model 2	0.484	(0.379, 0.618)	<0.001
Model 3	0.415	(0.305, 0.563)	<0.001

Model 1: unadjusted. Model 2: Adjusted for age, sex, BMI, ASA, smoking history, alcohol consumption history, education level, MoCA, and preoperative comorbidities. Model 3: Adjusted for all covariates in Model 2 plus type of surgery, operative time, preoperative inflammatory markers, preoperative hemoglobin, preoperative renal function (Cr, BUN), intraoperative blood loss, intraoperative transfusion, intraoperative hypotension, intraoperative and postoperative opioid (sufentanil and remifentanil) consumption, and postoperative 24-h NRS score. OR, odds ratio; CI, confidence interval; TXA group, intravenous tranexamic acid group; control group, no intravenous tranexamic acid group.

### 3.4 Association between postoperative inflammatory markers and POD

In the fully adjusted regression model, the results showed that patients with POD exhibited a higher inflammatory tendency across all markers except for MLR and PLR. Each one-unit increase in NLR was associated with a 1.09-fold increase in the risk of POD [95% CI: (1.058, 1.122), *p* < 0.001]. Four other inflammatory markers also demonstrated statistical significance: SIRI [1.052 (1.022, 1.083), *p* < 0.001], SII [1.000 (1.000, 1.000), *p* < 0.001], hs-CRP [1.012 (1.007, 1.017), *p* < 0.001], and IL-6 [1.003 (1.000, 1.005), *p* = 0.017] (Table 4).

**Table 4:** Association between postoperative inflammatory markers and postoperative delirium.

Variables	OR	95% CI	*P*
NLR			
Model 1	1.082	(1.058, 1.106)	<0.001
Model 2	1.078	(1.054, 1.102)	<0.001
Model 3	1.090	(1.058, 1.122)	<0.001
SIRI			
Model 1	1.055	(1.028, 1.082)	<0.001
Model 2	1.052	(1.025, 1.079)	<0.001
Model 3	1.052	(1.022, 1.083)	<0.001
MLR			
Model 1	1.058	(0.907, 1.234)	0.471
Model 2	1.058	(0.903, 1.240)	0.484
Model 3	1.039	(0.861, 1.253)	0.691
SII			
Model 1	1	(1.000, 1.000)	<0.001
Model 2	1	(1.000, 1.000)	<0.001
Model 3	1	(1.000, 1.000)	<0.001
PLR			
Model 1	1.001	(1.000, 1.002)	0.027
Model 2	1.001	(1.000, 1.002)	0.072
Model 3	1.001	(0.999, 1.002)	0.273
hs-CRP			
Model 1	1.014	(1.010, 1.017)	<0.001
Model 2	1.013	(1.009, 1.017)	<0.001
Model 3	1.012	(1.007, 1.017)	<0.001
IL-6			
Model 1	1.003	(1.002, 1.005)	<0.001
Model 2	1.003	(1.002, 1.005)	<0.001
Model 3	1.003	(1.000, 1.005)	0.017

Model 1: unadjusted. Model 2: Adjusted for age, sex, BMI, ASA, smoking history, alcohol consumption history, education level, MoCA, and preoperative comorbidities. Model 3: Adjusted for all covariates in Model 2 plus type of surgery, operative time, preoperative inflammatory markers, preoperative hemoglobin, preoperative renal function (Cr, BUN), intraoperative blood loss, intraoperative transfusion, intraoperative hypotension, intraoperative and postoperative opioid (sufentanil and remifentanil) consumption, and postoperative 24-h NRS score. NLR, neutrophil-to-lymphocyte ratio; SIRI, systemic inflammation response index; MLR, monocyte-to-lymphocyte ratio; SII, systemic immune-inflammation index; PLR, platelet-to-lymphocyte ratio; hs-CRP, high-sensitivity C-reactive protein; IL-6, interleukin-6; OR, odds ratio; CI, confidence interval; TXA group, intravenous tranexamic acid group; Control group, no intravenous tranexamic acid group.

### 3.5 Association between TXA and inflammatory markers

To evaluate the association between TXA administration and postoperative inflammatory markers, linear regression analysis was performed to determine the *β* (95% CI). In Model 1, TXA administration was significantly associated with all postoperative inflammatory markers. The associations between TXA use and postoperative inflammatory markers remained consistent after adjustment for covariates including age, sex, BMI, ASA classification, smoking history, alcohol consumption history, education level, MoCA score, preoperative comorbidities, type of surgery, operative duration, preoperative inflammatory marker levels, perioperative Hb levels, preoperative renal function, and other covariates (Table 5).

**Table 5:** Association between tranexamic acid and postoperative inflammatory markers.

Variables	*β*	95% CI	*P*
NLR			
Model 1	−2.194	(−2.828, −1.560)	<0.001
Model 2	−2.162	(−2.795, −1.529)	<0.001
Model 3	−2.153	(−2.786, −1.521)	<0.001
SIRI			
Model 1	−1.590	(−2.191, −0.990)	<0.001
Model 2	−1.641	(−2.239, −1.043)	<0.001
Model 3	−1.715	(−2.322, −1.109)	<0.001
MLR			
Model 1	−0.104	(−0.187, −0.022)	0.013
Model 2	−0.122	(−0.195, −0.029)	0.008
Model 3	−0.118	(−0.203, −0.034)	0.006
SII			
Model 1	−547.591	(−740.926, −354.255)	<0.001
Model 2	−535.464	(−729.726, −341.203)	<0.001
Model 3	−540.082	(−736.782, −343.382)	<0.001
PLR			
Model 1	−28.130	(−40.616, −15.645)	<0.001
Model 2	−25.872	(−38.347, −13.398)	<0.001
Model 3	−26.755	(−38.705, −13.609)	<0.001
hs-CRP			
Model 1	−11.375	(−15.117, −7.663)	<0.001
Model 2	−11.018	(−14.742, −7.294)	<0.001
Model 3	−11.038	(−14.685, −7.392)	<0.001
IL-6			
Model 1	−17.126	(−24.606, −9.646)	<0.001
Model 2	−16.395	(−23.819, −8.971)	<0.001
Model 3	−16.755	(−24.258, −9.253)	<0.001

Model 1: unadjusted. Model 2: Adjusted for age, sex, BMI, ASA, smoking history, alcohol consumption history, education level, MoCA, and preoperative comorbidities. Model 3: Adjusted for all covariates in Model 2 plus type of surgery, operative time, preoperative inflammatory markers, preoperative hemoglobin, preoperative renal function (Cr, BUN), intraoperative blood loss, intraoperative transfusion, intraoperative hypotension, intraoperative and postoperative opioid (sufentanil and remifentanil) consumption, and postoperative 24-h NRS score. NLR, neutrophil-to-lymphocyte ratio; SIRI, systemic inflammation response index; MLR, monocyte-to-lymphocyte ratio; SII, systemic immune-inflammation index; PLR, platelet-to-lymphocyte ratio; hs-CRP, high-sensitivity C-reactive protein; IL-6, interleukin-6; CI, confidence interval; TXA group, intravenous tranexamic acid group; control group, no intravenous tranexamic acid group.

### 3.6 Mediation analysis

**Figure 2:**
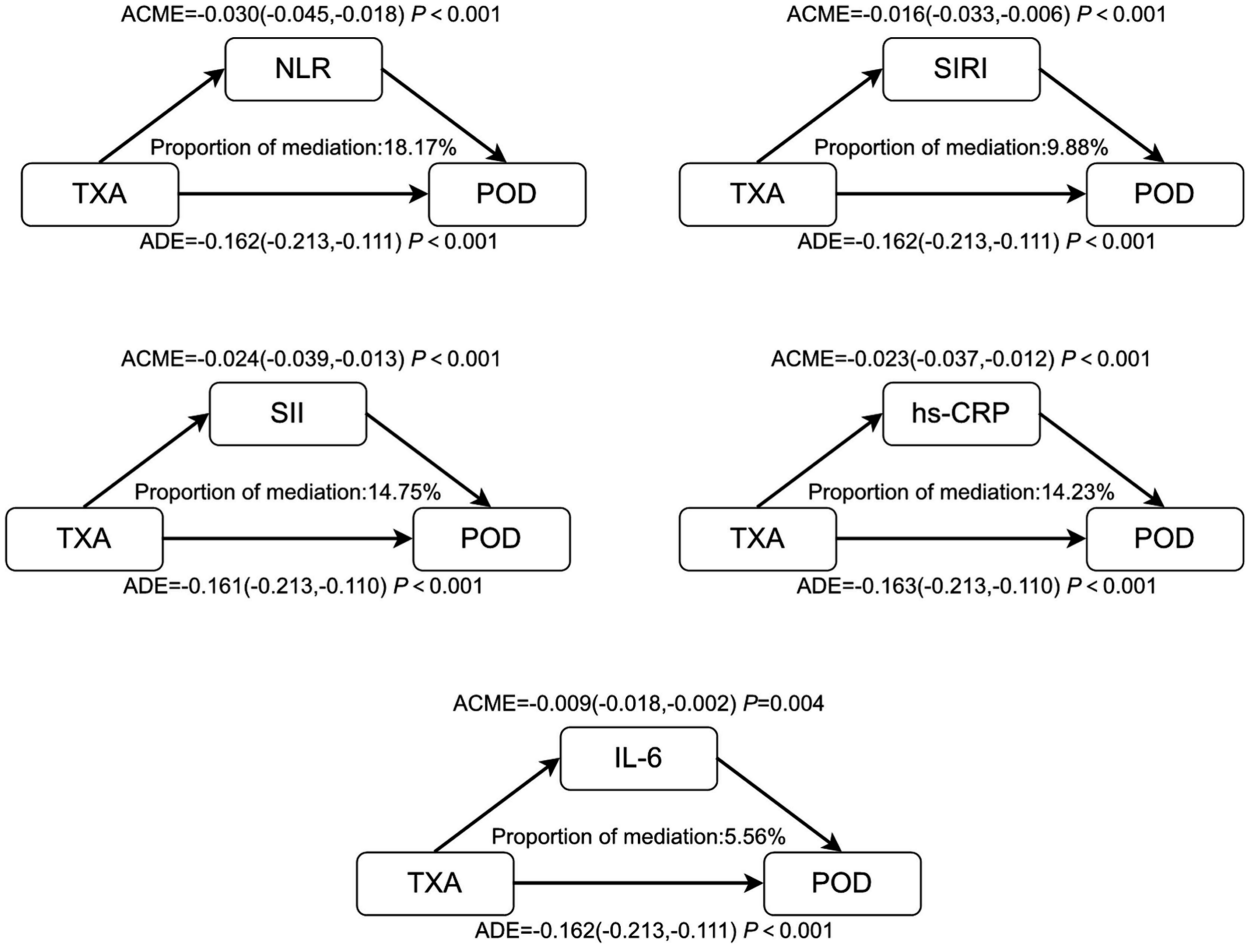
Mediating effect of postoperative inflammatory markers on the association between tranexamic acid and postoperative delirium. ACME, average causal mediation effect; ADE, average direct effect; NLR, neutrophil-to-lymphocyte ratio; SIRI, systemic inflammation response index; SII, systemic immune-inflammation index; hs-CRP, high-sensitivity C-reactive protein; IL-6, interleukin-6; TXA, tranexamic acid; POD, postoperative delirium.

We performed exploratory mediation analyses for five inflammatory markers, namely, NLR, SIRI, SII, hs-CRP, and IL-6, to investigate their potential mediating roles in the association between TXA administration and POD. After adjusting for potential confounders based on Model 3, significant mediation effects were observed for NLR (ACME = −0.030, *p* < 0.001, proportion mediated = 18.17%), SIRI (ACME = −0.016, *p* < 0.001 proportion mediated = 9.88%), SII (ACME = −0.024, *p* < 0.001, proportion mediated = 14.75%), hs-CRP (ACME = −0.023, *p* < 0.001, proportion mediated = 14.23%), and IL-6 (ACME = −0.009, *p* = 0.004, proportion mediated = 5.56%). The detailed results are presented in Figure 2.

### 3.7 Subgroup and interaction analyses

**Figure 3:**
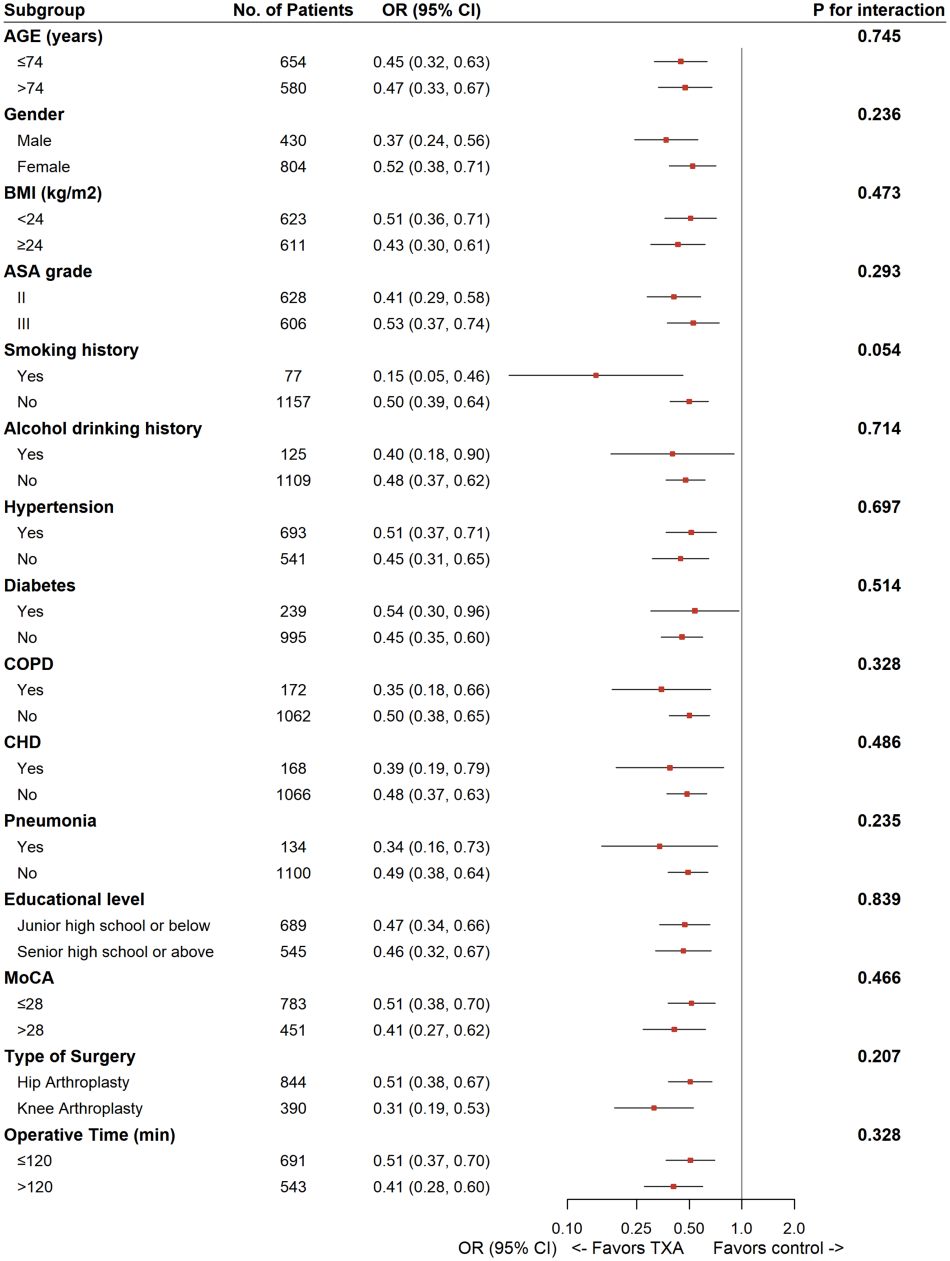
Subgroup analysis of the association between tranexamic acid and postoperative delirium in elderly patients undergoing hip or knee arthroplasty. BMI, body mass index; ASA, American Society of Anesthesiologists; CHD, coronary heart disease; COPD, chronic obstructive pulmonary disease; MoCA, Montreal Cognitive Assessment; OR, odds ratio; CI, confidence interval.

To further verify the consistency of our findings, stratified analyses were performed based on baseline characteristics. Stratification variables included the following: gender (male vs. female), age (≤74 vs. >74 years), BMI (<24 vs. ≥24 kg/m^2^), smoking history (yes vs. no), alcohol consumption history (yes vs. no), preoperative comorbidities (yes vs. no), educational level (junior high school or below vs. senior high school or above), MoCA score (≤28 vs. >28), ASA grade (II vs. III), type of surgery (hip vs. knee arthroplasty), and operative time (≤120 vs. >120 min). The results demonstrated that the association between intravenous TXA administration and POD incidence remained significant across all subgroups (Figure 3). The *p*-values for interaction were not statistically significant across all stratification variables (P for interaction >0.05), suggesting that the association between TXA administration and a lower risk of POD was generally consistent across different subgroups.

## 4 Discussion

This study analyzed data from 1,234 patients aged 65 years and older undergoing hip or knee arthroplasty to explore the association between TXA and the occurrence of POD and to determine whether this relationship is mediated by inflammatory markers. Our findings demonstrate that compared with the control group, patients in the TXA group had a significantly lower risk of POD. After controlling for potential confounders—including age, gender, BMI, ASA grade, smoking history, alcohol consumption history, preoperative comorbidities, and MoCA score—intravenous TXA administration remained a robust protective factor against POD (OR = 0.415; 95% CI, 0.305–0.563, *p* < 0.001). Furthermore, mediation analysis revealed that NLR, SIRI, SII, hs-CRP, and IL-6 partially mediated the association between TXA and POD. Subgroup analyses further confirmed the generalizability of this protective effect across diverse elderly populations.

Postoperative delirium is a common perioperative complication among the elderly, primarily characterized by acute and fluctuating disturbances in attention and awareness. In this study, we utilized CAM as the diagnostic standard (23). Results showed that the incidence of POD among elderly patients undergoing hip or knee arthroplasty was 33.5%, which is consistent with the previously reported range of 13.0 to 55.9% (24). Currently, the neuroinflammation hypothesis is recognized as a critical mechanism underlying the development of POD. Surgical trauma, anesthesia, and perioperative stress can activate the peripheral immune system, leading to the release of pro-inflammatory cytokines that traverse the blood–brain barrier. Once these peripheral signals enter the central nervous system, they activate microglia and trigger an intracerebral inflammatory cascade, ultimately resulting in neuronal injury and synaptic transmission dysfunction. A study involving human brain autopsies revealed that the activation levels of microglia and astrocytes were significantly higher in patients with delirium than in those without, supporting the neuroinflammatory mechanism of delirium (25). Current clinical evidence also demonstrates that peripheral inflammatory levels in the early postoperative period are significantly higher in patients with POD than in those without (26). Specifically, the abnormal elevation of markers such as NLR, SIRI, CRP, and IL-6 has been confirmed as an independent risk factor for the development of POD (16, 17, 27). The regression analysis in our study demonstrated that elevated levels of NLR, SIRI, MLR, SII, PLR, hs-CRP, and IL-6 were significantly and positively correlated with the occurrence of POD. These findings are consistent with previous reports and further emphasize the pivotal role of the inflammatory response in the development and progression of POD.

Beyond its primary role as an antifibrinolytic agent for hemostasis, TXA also exhibits distinct anti-inflammatory properties. Our study found that patients who received intravenous TXA demonstrated a significantly attenuated postoperative inflammatory response. Although fibrinolysis and inflammation are independent processes in many respects, evidence suggests a tight interplay between the two systems. Inflammation can induce hyperfibrinolysis, while activated plasmin, in turn, promotes the release of inflammatory mediators, thereby establishing a vicious cycle (28, 29). Consequently, the inhibition of plasmin formation by TXA may contribute to the prevention of excessive inflammation. The anti-inflammatory effects of TXA have been extensively validated in both basic and clinical research. At the transcriptomic level, a study by Alexander F. L. Later et al. investigated the mRNA expression levels of 114 inflammatory genes before and after surgery, finding that patients treated with TXA exhibited less upregulation of pro-inflammatory genes and greater upregulation of anti-inflammatory genes (30). At the cellular pathway level, Xiaowu Wu et al. utilized a rat model of multiple trauma and demonstrated that TXA can reduce inflammatory parameters such as neutrophils and macrophages while inhibiting the elevation of neutrophil elastase, myeloperoxidase, and complement C5a levels (31). Furthermore, clinical studies conducted in orthopedic surgery and cardiac surgery have demonstrated that the administration of TXA can effectively reduce postoperative inflammatory levels (11, 32). Another meta-analysis of hip and knee arthroplasty revealed that patients receiving high-dose TXA had lower postoperative levels of IL-6 and CRP compared to those receiving lower doses, with no significant difference in the incidence of thromboembolic events (33). This not only supports the safety and efficacy of TXA in clinical practice but also suggests that its modulation of the inflammatory response may be dose-dependent, warranting further in-depth exploration in future studies.

To further elucidate the association between TXA administration and the occurrence of POD in elderly patients undergoing hip or knee arthroplasty, we performed an exploratory mediation analysis. Mediation analysis revealed that postoperative inflammatory markers partially mediated the association between TXA and POD. Among these markers, NLR, SII, and hs-CRP showed relatively higher mediation proportions of 18.17, 14.75, and 14.23%, respectively, suggesting a potential trend toward mediating roles. Notably, in the fully adjusted model, MLR and PLR were not significantly associated with POD. This finding may suggest that acute postoperative neuroinflammation is more closely related to neutrophil-associated inflammatory pathways than to monocyte- or platelet-related inflammatory responses. Regarding the selection of inflammatory markers, in addition to conventional assays for hs-CRP and IL-6, we focused specifically on blood cell-based composite inflammatory indices, such as NLR, SIRI, MLR, SII, and PLR. These parameters, derived from routine blood tests, are not only cost-effective and easy to implement clinically but also demonstrate high reproducibility. By integrating information from different types of immune cells, these indices overcome the limitations of single markers and provide a more comprehensive and stable assessment of systemic inflammation. NF-κB is a central signaling pathway in the inflammatory response and is extensively involved in immune regulation, cellular stress, and the production of inflammatory mediators (34). Surgical trauma or infection can trigger a systemic inflammatory response, leading to the increased release of pro-inflammatory cytokines such as IL-1β, IL-6, and tumor necrosis factor-*α* (TNF-α) (14). The release of these inflammatory factors can, in turn, activate the NF-κB pathway through positive feedback, further amplifying the inflammatory response (34, 35). It is well-established that inflammatory levels are significant risk factors for neurocognitive dysfunction. A study by Xu et al. confirmed that prolonged anesthesia can activate the NF-κB pathway, driving the upregulation of inflammatory cytokines and exacerbating microglial activation, which subsequently leads to neurobehavioral changes (36, 37). This underscores the pivotal role of NF-κB in the pathogenesis of postoperative neurocognitive disorders. Similarly, Wenyi Liu et al. suggested that modulating the NF-κB inflammatory pathway could serve as a potential neuroprotective mechanism to prevent postoperative delirium (38). Importantly, several basic studies have confirmed that TXA possesses pharmacological activity that inhibits the NF-κB pathway. In both bone fracture trauma and hemorrhagic shock rat models, TXA has demonstrated significant anti-inflammatory activity. The underlying mechanism primarily involves the downregulation of the NF-κB signaling pathway, which in turn reduces the release of downstream pro-inflammatory cytokines (39, 40). The TXA group exhibited lower postoperative inflammatory marker levels and a reduced incidence of POD. Drawing on mediation analysis and prior literature, the protective effect of TXA against POD may be partially attributable to its modulation of the systemic inflammatory response. Although direct assessment of cellular signaling pathways was not performed in the present study, the aforementioned evidence raises the possibility that TXA suppresses the NF-κB pathway-mediated inflammatory cascade, a potential mechanism that warrants validation through future basic research.

Although our study benefits from a large sample size and rigorous methodology, several limitations must be acknowledged. First, constrained by the retrospective design and clinical realities such as the patients’ financial burden and routine testing protocols, this study did not routinely monitor other specific inflammatory markers, such as TNF–*α* or erythrocyte sedimentation rate (ESR). We focused exclusively on early inflammatory levels within the first 48 h postoperatively, lacking longitudinal tracking of long-term inflammatory dynamics. Second, as a retrospective study, the intraoperative use of TXA was not based on prospective randomized allocation but was primarily determined by the surgeon’s clinical practice preferences and individualized assessment of each patient’s bleeding and thrombotic risks. This “real-world” decision-making process, while reflective of the complexity inherent in clinical practice, inevitably introduced selection bias and heterogeneity in TXA administration triggers. Although major clinical variables were controlled through multivariable regression analysis, unmeasured potential confounders, such as individual surgeon experience and other factors, may still have exerted a residual influence on the findings. Third, this was a single-center study, and all patients were recruited from the same medical institution, where anesthesia and perioperative management protocols were relatively consistent. Because the findings were not validated in an external cohort, the generalizability of our conclusions to other populations and healthcare settings may be limited. Fourth, inflammatory markers were measured within 48 h after surgery, whereas POD was assessed within 7 days after surgery. This design may introduce inherent methodological limitations regarding the absolute temporal and causal sequence; therefore, this analysis should be considered exploratory. In addition, POD was assessed using CAM based on retrospective electronic medical record documentation, which depended on the quality of the medical records at that time and may have led to an underestimation of hypoactive delirium. Furthermore, the present study focused primarily on the mediating role of systemic inflammatory response in the association between TXA and POD, without further exploring other potential underlying mechanisms. Given these limitations, future multicenter, large-sample, prospective randomized controlled trials with more comprehensive baseline and inflammatory markers and longer follow-up periods are warranted to further investigate the association between TXA administration and POD, as well as the underlying mechanisms.

## 5 Conclusion

This retrospective study of elderly patients undergoing hip or knee arthroplasty demonstrates that the intravenous administration of TXA is a protective factor against the occurrence of POD. The mediation analysis suggested that the potential protective association of TXA administration with POD may be partially explained by perioperative inflammatory responses. By understanding these relationships, we can develop more effective methods and strategies to improve the quality of postoperative recovery in elderly patients.

## Data Availability

The data analyzed in this study is subject to the following licenses/restrictions: the application for the original data of this study requires the consent of the corresponding author and other researchers. Requests to access these datasets should be directed to Junyang Chu, 13855127642@163.com.
